# MULTIDIMENSIONAL CONSTRUCTS OF SLEEP IN OLDER ADULTS

**DOI:** 10.1093/geroni/igac059.1173

**Published:** 2022-12-20

**Authors:** Christopher Kaufmann, Katie Stone

## Abstract

Sleep disturbances are common in older adults and are associated with a variety of adverse mental and physical health outcomes. While most studies have characterized sleep based on a single dimension of sleep (typically sleep duration or self-reported sleep quality), there is a growing paradigm shift to characterize “Sleep Health” as multidimensional. More research is needed to identify the clinical utility of a multidimensional approach to characterizing sleep specifically in older adults. In this symposium, we will present five studies that explore how multidimensional Sleep Health relates to age-related health outcomes (e.g., fall risk). Our first presentation will identify associations between subjective/objective sleep quality and physical activity, and examine the moderating role of chronotype. Second, we will examine the extent to which a multidimensional index of Sleep Health and its individual dimensions relate to fall risk. Third, we will ascertain the impact of mindfulness on various Sleep Health dimensions. Fourth, we will identify the extent to which discordance in subjective and objective sleep metrics are related to cognitive functioning. Finally, we will explore how the accumulation of disturbances in Sleep Health may increase risk for early mortality. Findings from this symposium will highlight the clinical relevance of a multidimensional approach to Sleep Health in older adults, and identify the ways in which sleep interventions can be tailored to specific sleep dimensions to promote health in later life.

